# Safeguarding Athletes Against Head Injuries Through Advances in Technology: A Scoping Review of the Uses of Machine Learning in the Management of Sports-Related Concussion

**DOI:** 10.3389/fspor.2022.837643

**Published:** 2022-04-20

**Authors:** Anne Tjønndal, Stian Røsten

**Affiliations:** Department of Leadership and Innovation, Faculty of Social Sciences, Nord University, Bodø, Norway

**Keywords:** machine learning, sports-related concussion (SRC), deep learning, athlete welfare, sport injury prevention, sport technologies, sport and health

## Abstract

Sports injury prevention is an important part of the athlete welfare and safeguarding research field. In sports injury prevention, sport-related concussion (SRC) has proved to be one of the most difficult and complex injuries to manage in terms of prevention, diagnosis, classification, treatment and rehabilitation. SRC can cause long-term health issues and is a commonly reported injury in both adult and youth athletes around the world. Despite increased knowledge of the prevalence of SRC, very few tools are available for diagnosing SRC in athletic settings. Recent technological innovations have resulted in different machine learning and deep learning methodologies being tested to improve the management of this complex sports injury. The purpose of this article is to summarize and map the existing research literature on the use of machine learning in the management of SRC, ascertain where there are gaps in the existing research and identify recommendations for future research. This is explored through a scoping review. A systematic search in the three electronic databases SPORTDiscus, PubMed and Scopus identified an initial 522 studies, of which 24 were included in the final review, the majority of which focused on machine learning for the prediction and prevention of SRC (*N* = 10), or machine learning for the diagnosis and classification of SRC (*N* = 11). Only 3 studies explored machine learning approaches for the treatment and rehabilitation of SRC. A main finding is that current research highlights promising practical uses (e.g., more accurate and rapid injury assessment or return-to-sport participation criteria) of machine learning in the management of SRC. The review also revealed a narrow research focus in the existing literature. As current research is primarily conducted on male adolescents or adults from team sports in North America there is an urgent need to include wider demographics in more diverse samples and sports contexts in the machine learning algorithms. If research datasets continue to be based on narrow samples of athletes, the development of any new diagnostic and predictive tools for SRC emerging from this research will be at risk. Today, these risks appear to mainly affect the health and safety of female athletes.

## Introduction

Safeguarding and athlete welfare in sport have received increased public and academic attention in the last decade (Nery et al., 2021). Together, these terms encompass health and safety issues related to topics such as gendered violence (Barker-Ruchti et al., 2021), abusive coaching practices (McMahon et al., 2020), racism (Manning, 2021), bullying (Parent et al., 2021), the legitimization and stigmatization of disordered eating (Papathomas, 2019), psychological abuse (Krahn, 2021) and the risk of sustaining sports injuries (White, 2021). In the context of sports injuries, sports-related concussion (SRC) represents an important subject in that it can result in long-term health issues for athletes (Gagnon and Ptito, 2017; Sindelar and Bailes, 2017). The Concussion in Sport Group (CISG) defines SRC as: “a traumatic brain injury that is defined as a complex pathophysiological process affecting the brain, induced by biomechanical forces with several common features that help define its nature” (McCrory et al., 2017, p. 877).

For coaches and athletes, SRCs are notoriously difficult injuries to manage, as the changes in the neurological function associated with concussion are often rapid and resolved spontaneously (Malcolm, 2020). Due to this, many SRCs can go unreported and unrecognized by the athletes themselves and their coaches (Ventresca and McDonald, 2020). The difficulty in detecting this type of injury is part of the reason why SRC has been called “a silent epidemic” (Carroll and David, 2012) and “a contemporary crisis in sport” (Malcolm, 2020). There are also cases where SRC is purposely left unreported, as contracting a concussion will lead to athletes being suspended for set periods of time in many sports. Therefore, coaches and athletes may avoid reporting head injuries if they suspect a concussion, fearing that it will result in the athlete being omitted from important competitions. These complexities showcase how all studies on the topic of SRC concern important aspects of athlete safety and health in both adult and youth sport (Tjønndal et al., 2022).

SRC is a commonly reported injury among adult and youth athletes around the globe and represents a safeguarding issue in contact and non-contact sports at the elite and recreational levels (Tjønndal and Wågan, 2021). Ferris et al. (2021, p. 1040–1041) state that “up to an estimated 3.8 million sport-related [...] concussions occur annually in the United States” alone. Clinically, a concussion is known to affect an athlete's memory, reaction time and balance. A previous concussion may also lead to athletes being prone to subsequent concussions, which could result in cognitive impairment, depression or chronic traumatic encephalopathy (Ventresca and McDonald, 2020). As a research field, SRC has expanded significantly over the last 20 years. This is reflected in the growth of systematic reviews of different sports and age groups (e.g., Langlois et al., 2006; Clay et al., 2013; Pfister et al., 2016), as well as sport-specific reviews (Gardner et al., 2015; Mooney et al., 2020; Tjønndal et al., 2022), reviews on gender differences (Dick, 2009), risk factors (Abrahams et al., 2014), the effect of protective sports equipment (Benson et al., 2009) and the use of wearable technologies in the detection of SRC (Schmid et al., 2021). These studies and others reflect an increased research interest in the management of SRC. In this context, we use the term management to describe all aspects of SRC, including the prediction, prevention, diagnosis, classification, treatment and rehabilitation of the injury. Arguably, the growth of research on SRC is a response to increased public awareness of the risks of SRC and is a part of a wider movement of securing athlete welfare in sport (Graham et al., 2014).

While the incidence of SRC may vary between different sports, it is difficult to assess exactly how common SRC is in sport and among different age groups of athletes (McCrory, 2013). For instance, in a systematic review of head injuries and concussion in boxing, the studies included in the review reported between 10 and 70% head injuries of all the recorded injuries in the athlete populations (Tjønndal et al., 2022). In another recent systematic review of the prevalence of SRC across 12 youth sports, Pfister et al. (2016) estimated that the overall incidence rate of SRC was 0.23 per 1000 athlete exposures (AEs), varying from the highest in rugby (4.18) and ice hockey (1.20) and the lowest in volleyball (0.03) and baseball (0.07). These reviews showcase that SRC is a complicated area of study in the field of athlete welfare and safeguarding.

Despite the increased knowledge of the prevalence of SRC, very few tools are available to immediately diagnose or measure SRC in athletic settings. A commonly used diagnosis protocol for SRC is the Sideline Concussion Assessment Tool (SCAT), which combines both subjective and objective measurements and takes between 10 and 20 minutes to conduct (Yengo-Kahn et al., 2016; Echemendia et al., 2017). However, recent technological innovations have resulted in different machine learning and deep learning methodologies being tested to improve injury prediction, prevention and diagnosis in sports (Jauhiainen et al., 2021; Van Eetvelde et al., 2021).

Machine learning is a field of computer science that automates analytical model building to reveal patterns in data to help humans make better decisions (Cenek et al., 2018). Similarly, deep learning is described as a class of artificial neural networks that learn in an unsupervised manner. In health care, these approaches are used to analyse medical data in disease prevention, diagnosis, patient monitoring and the development of new protocols (Patel et al., 2021). Machine learning approaches can recognize patterns, trends and associations and are critical for understanding SRC (Agoston, 2019). The emergence of machine learning algorithms in medical practice and their capacity to integrate thousands of biomarkers to build objective and automated *in vivo* detection tools to support clinical diagnosis of SRC is a promising development (Jordan and Mitchell, 2015; Obermeyer and Emanuel, 2016). Put simply, the use of machine learning techniques in sports injury prevention could provide faster and more accurate ways of managing SRC in athletes in a wide range of sports.

From this starting point, the purpose of the review is to: (1) summarize and map the existing research literature on the use of machine learning in the management of SRC and (2) ascertain where there are gaps in the existing research and identify recommendations for future research. We investigate these research purposes through a scoping review of research literature using the following three relevant academic databases: SPORTDiscus, PubMed and Scopus.

## Method: Scoping Review

If we use the analogy of an ocean to express the current body of knowledge on machine learning in the management of SRC, at present there are no academic reviews to tell us: (a) the size or shape of the ocean (i.e., prevalence), (b) in which direction the wind blows and the waves travel (i.e., potentials for use) or (c) which species (i.e., sports) are present in the ocean. As machine learning in the management of SRC is a new scholarly field, a scoping review is valuable for gaining an overview of the research that has been conducted on this topic (Arksey and O'Malley, 2005). While scoping reviews do not typically include evidence-based methodological processes [e.g., assessment of bias, rating of level of evidence (LoE)] as recommended in the statement guidelines for the Preferred Reporting Items for Systematic Reviews and Meta-Analyses (PRISMA) (Tricco et al., 2018), they are particularly helpful for understanding complex topics by appraising literature with a wide range of different study designs and methods (Peters et al., 2020). As SRC is often described as one of the most complex injuries of the most complex organ (Agoston, 2019), we find the scoping review to be a fruitful methodological approach for our examination of the field.

According to Arksey and O'Malley (2005), a scoping review is ideal for investigating the character and extent of research in a particular domain or on a specific topic. The present review was designed to summarize the current body of knowledge on how machine learning is used in the context of managing SRC. We chose a scoping review approach because the use of machine learning for the management of SRC is a relatively new topic in the sports injury field. As Bergeron et al. (2019, p. 1363) state: “the advent of using machine learning in addressing the complexity of various human health challenges is only recently underway.” Therefore, a scoping review is regarded as an appropriate approach for our examination, in that it is a review method that often precedes systematic literature reviews and is frequently used to map novel research fields to identify research gaps requiring further attention (Munn et al., 2018; Peters et al., 2020). In the following, we present our search strategy, the procedure of the scoping review and the study's limitations.

### Search Strategy

The literature search was guided by the PRISMA-ScR guidelines (Tricco et al., 2018) and was conducted in October 2021 in the three academic databases of SPORTDiscus, PubMed and Scopus. These databases were chosen because they collectively provide a comprehensive academic profile of machine learning in the management of SRC in a wide variety of scholarly disciplines (e.g., sports science, medicine, biomedical, neurology, engineering, sociology, pedagogy, psychology).

The studies to be considered for inclusion in the review were identified using the following search term: “machine learning” or “ml” or “artificial intelligence” or “ai” or “learning algorithms” or “learning systems” AND “concussion” or “head injury” or “mild traumatic brain injury” or “mild tbi” or “mtbi” AND “sports” or “athletics” or “athletes” or “players” or “sport” or “competition.” No restrictions on publications dates were made in the search.

Although the intention was to include a wide profile of research on machine learning in the management of SRC, some eligibility criteria were applied. For inclusion in the review, studies needed to (1) focus on machine learning approaches to the management of SRC, (2) include data from athletes and/or be original empirical studies conducted in the context of sport, (3) be reported in peer-reviewed journals and (4) be written in English.

### Procedure of the Review

**Figure 1:**
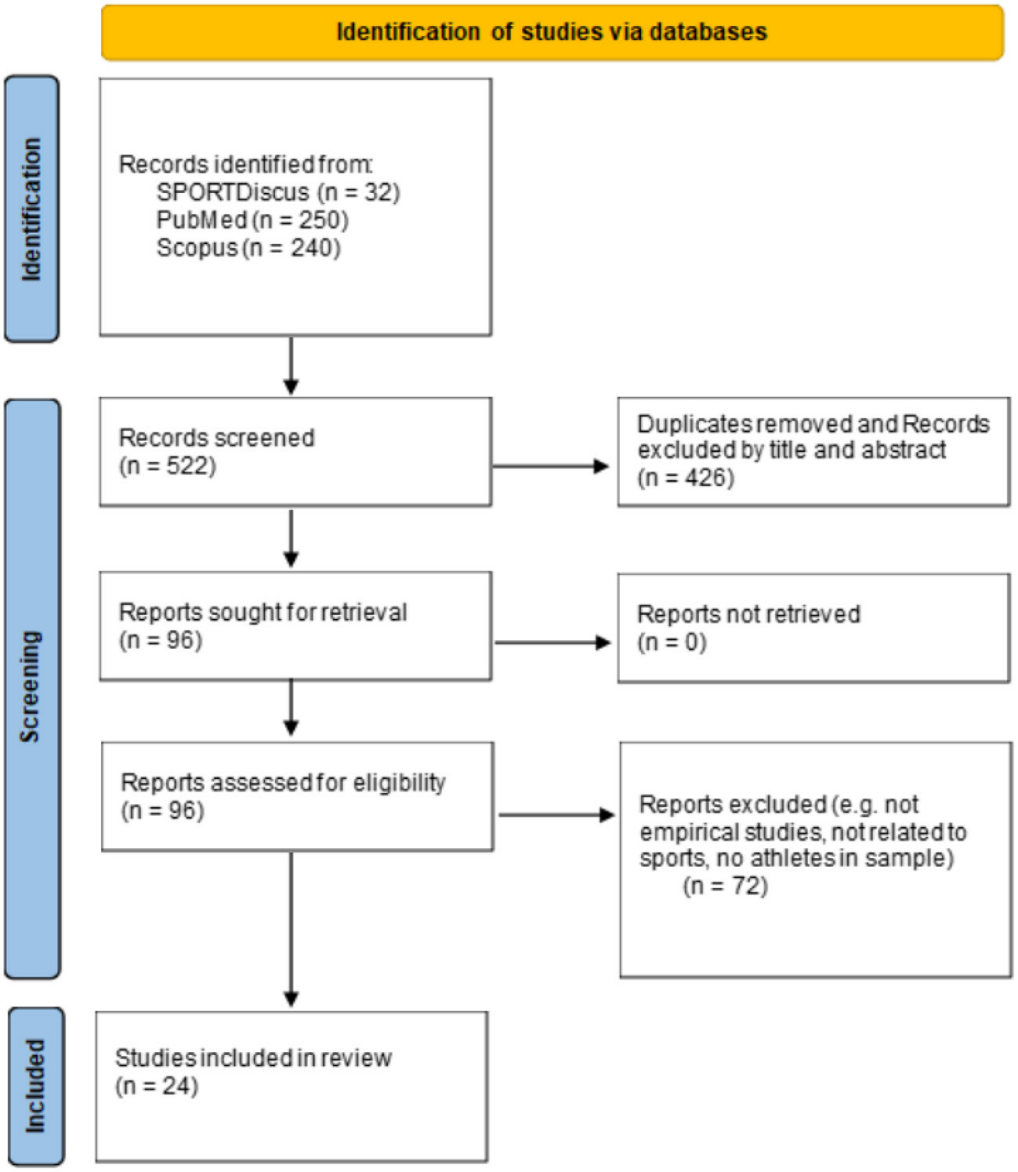
Flow chart of the scoping review process, adapted from Page et al. (2021).

The initial search identified 522 articles. Two hundred forty articles were identified from Scopus, 250 via PubMed and 32 through SPORTDiscus (Figure 1). The process for identifying articles for inclusion in the final review can be divided into two main phases. In phase 1, each individual study's author(s) and year of publication were recorded schematically. From this initial list of entries, the second author screened the title and abstract to identify relevant studies against the inclusion criteria. This preliminary phase revealed that many of the identified articles did not match the review's aim or fulfill the inclusion criteria. For example, a large number of hits were related to contexts other than sport (e.g., hospital, traffic injuries), utilized animal samples (e.g., mice, rats, piglets), or investigated the “phenomenon” of SRC but did not consider the use of machine learning approaches in its management. In those instances where the second author was uncertain about including an article in the potential final sample, the first author was consulted. In phase 1, duplicates between the three databases were removed.

After the initial screening of titles and abstracts, 426 studies were excluded, leaving a total of 96 studies for further analysis. From this refined list, a more comprehensive assessment took place in phase 2, where both authors performed the full text screening of all the 96 articles. When assessing the full texts for eligibility, it was apparent that several articles only mentioned machine learning briefly but did not directly apply machine learning to analyse SRC or present the findings from such an approach. These articles were therefore excluded as they did not meet the inclusion criteria. In addition, several studies were considered according to the study sample or the use of non-sporting datasets (e.g., to test/simulate effects of different head impacts). For instance, in cases with both athletes (SRC) and other concussion patients, at least 50% of subjects had to have sustained their concussion in a sporting context in order for the study to be included in the review. Any uncertainties or issues that occurred during the process were resolved in discussions between the two authors. After the full text screening, 24 studies were ultimately included in the final review. The process of the scoping review is illustrated in Figure 1.

Once the final list had been completed, we summarized the following information about the included articles: year of publication, title of the journal, participants (sex and age), sample size, sports, geographical region the study was conducted in, study design and methods, name of the machine learning algorithm, key findings and how the machine learning approaches were used in the management of SRC (three categorisations: (1) diagnosis and classification, (2) prediction and prevention and (3) treatment and rehabilitation). The first author reviewed and amalgamated the work, while any disagreements were discussed. This clarified our understanding of the studies and was valuable to ensure consistency.

### Limitations

Although this review provides insight into how machine learning can be used to manage SRC in different ways, there are limitations in our methodological approach. An important limitation is that both authors are social scientists and that this topic is dominated by research on medicine and engineering. When discussing our interpretations of the different studies, working in a dyad rather than a larger group of researchers may also have been a limitation. For instance, being able to discuss the studies included in the review with researchers in the field of sports medicine may have been more productive. However, as this is not a systematic review (and therefore does not aim to evaluate LoE), we do not consider our social scientific backgrounds to be detrimental to our analysis and findings. Finally, as the review was restricted to peer-reviewed studies written in English we excluded research reported in other languages that may have been relevant to the topic. However, this was a pragmatic necessity, given that the authors are only familiar with English and the Scandinavian languages.

## Results

The presentation of the results is guided by the first purpose of this scoping review, which is to summarize and map the existing research literature on the use of machine learning in the management of SRC. The scoping review of the databases SPORTDiscus, PubMed and Scopus identified an initial 522 peer-reviewed studies reported in academic journals (see Figure 1). After applying the inclusion criteria to the titles and abstracts, 96 potentially relevant studies were included for full text examination by both authors. From these, 72 studies were excluded because they did not meet the inclusion criteria. There were two main reasons for exclusion at this stage: (1) no athletes were included in the sample (e.g., patients from non-sport related concussions) and (2) no machine learning techniques were applied in the study. This resulted in a final sample of 24 articles being included in our scoping review. The results of the scoping review are summarized in Table 1. Complete references of the included studies can be found in the reference list of this article.

**Table 1:** Results of the scoping review.

**References**	**Aim**	**Sample**	**Methods & measures**	**Findings**
Bazarian et al. (2021)	Validate classification accuracy of the concussion index in athletes.	*N* = 580 athletes (with and without concussion) Age: 13–25, Sports: football, soccer, basketball, lacrosse, rugby. Region: North America	Prospective cohort study between February 2017 and March 2019. Multimodal, EEG based Concussion Index including cognitive testing and symptom inventories. ML-method: The genetic algorithm.	Concussion index has high classification accuracy for identification of the likelihood of concussion at time of injury. Potential to aid in the clinical diagnosis of concussion and in the assessment of athletes' readiness to return to play.
Bergeron et al. (2019)	ML-approach to estimate symptom resolve time within 7, 14 and 28 days in high school athletes with SRC.	Data from the National Athletic Treatment, Injury and Network (NATION) injury surveillance program (2011–2014) from 147 high schools in 26 states. *N* **=** 1,611 concussion incidents. Sex: Male and female Age: N.R. (class year) Sports: football, wrestling, field hockey, basketball, soccer, lacrosse. Region: North America	Cohort study. Symptoms were recorded based on responses to an administered 17-item yes/no checklist. Created three distinct category thresholds of symptom resolution time; within 7, 14, and 28 d. ML-method: 10 classification algorithms considered (Naïve Bayes (NB), support vector machine (SVM), 5-nearest neighbors (5NN), C4.5 Decision Tree (C4.5D and C4.5N), Random Forest (RF100 and RF500), multilayer perceptron and radial basis function network).	ML demonstrated efficacy, while warranting further exploration, in developing symptom-based prediction models for practical estimation of SRC recovery in enhancing clinical decision support.
Bohsra et al. (2019)	Investigate the extent to which our present understanding of how mTBI affects brain activity and can be used to detect past concussions.	*N* = 39 male adults: 19 retired Canadian Football League athletes, the remaining 20 participants = control group. Sex: male Age: mean age athletes (57.6), mean age control group (53.7). Sports: football Region: North America	Three paradigm experiments. Self-report questionnaires, EEG Recording and pre-processing (Brain Vision Analyzer). ML-method: Support Vector Machines (SVMs).	A combination of statistics of single-subject ERPs and wavelet features yielded a classification accuracy of 81% with a sensitivity of 82% and a specificity of 80%, improving on current practice. The model was able to detect concussion effects in individuals who sustained their last injury as much as 45 years earlier.
Cai et al. (2018)	Develop an accurate and reliable injury predictor for concussion classification.	*N* = 58 cases from NFL to simulate the reconstructed head impacts using the Worcester Head Injury Model (WHIM). Age: Senior, Sports: football, Region: North America	Voxel-wise WM fiber strains from the brain as implicit features for concussion prediction ML-method: SVM and RF as baseline classifiers to benchmark the performance of deep learning. ML-method: deep learning network.	ML classifiers and deep learning outperformed all scalar injury metrics across all performance categories. These findings demonstrate the superior performances of deep learning in concussion prediction and suggest its promise for future applications in biomechanical investigations of TBI.
Cao et al. (2008)	Use ML to detect residual functional abnormalities at 30-day postinjury with multichannel EEG data.	*N* = 61 student athletes (27 M and 34 F). Age: 18–25. Sports: rugby and football. Region: North America	Multichannel EEG set under multiple conditions for assessments. ML-method: Support vector machine (SVM) to identify athletes who suffer from residual functional deficits after SRC.	SVM may be potentially used in clinical practice for automatic classification of athletes with residual brain functional abnormalities following a concussion episode.
Castellanos et al. (2021)	Develop a predictive model for sport-related concussion.	*N* = 15,628. Collegiate athletes (63%) and military cadets (37%). Age: 18–21 Sex: male and female Sports: baseball, basketball, cheerleading, cross country running, diving, track and field, field hockey, football, gymnastics, ice hockey, lacrosse, rowing, rugby, soccer, softball, swimming, tennis, volleyball, water polo, wrestling. Region: North America	Prospective design; Data from the CARE Consortium Study between the 2015 to 2016 academic year. Participants completed Level A (e.g., demographics, SCAT Symptom Checklist) and B (e.g., reaction time, quality of life) measures. Baseline data from 176 covariates including 957 features. ML-method: A linear support vector machine (SVM) to stratify subjects' risk for SRC based on baseline data.	The model identifies athletes and cadets who would go on to sustain SRC with comparable accuracy to many existing assessment tools and provides insights into potential risk and protective factors.
DiCesare et al. (2020)	Examine whether ML classification of sensor-recorded head impacts could produce a more accurate quantification of season-long sub-concussive head impacts (SCIs) exposure, and whether this would result in pronounced associations between SCI exposure and longitudinal changes to white matter (WM) microstructure.	*N* = 46 female athletes Age: Mean = 16, Sport: soccer. Region: North America	Prospective cohort study. Analyze; pre- and post-season MRI-scans (*N* = 22, control group), and video-verified head impact data (*N* = 24, training group). ML-method: XGBoost.	The ML classifier performed best and provided a superior means for removing spurious recordings and allowed for greater sensitivity in exploring and quantifying the relationship between SCI exposure and longitudinal changes of WM and head impacts.
Domel et al. (2021)	Present a deep learning algorithm (MiGNet) to differentiate between true and false head impacts for an instrumented mouthguard sensor (MiG2.0), and compare the predictive power between MigNet and support vector machine (SVM).	*N* = 66 (i.e., 12 collegiate and 49 high school athletes). Age: N.R. Sex: N.R. Sport: football. Region: North America	Cohort study. Measures; an instrumented mouthguard for measuring linear and angular head kinematics during impacts. Included video to verify head impacts (358 true and 500 false). ML-method: MigNet (a neural network classifier) and SVM.	MigNet (96% accuracy) perform better compared to SVM (91% accuracy).
Falcone et al. (2013)	Explore the feasibility of using speech analysis and ML for detecting whether an athlete is concussed.	*N* = 105 male athletes. Age: N.R.Sport: boxing. Region: N.R.	Cohort study over the course of pre-, during and post-assessments in a boxing tournament. Data from a mobile application test and based on speaking a fixed sequence of digits that appeared on screen every 1.5 s for 30 s. ML-method: Support Vector Machine (SVM).	Prediction results were verified against the diagnoses made by a ringside medical team and performance evaluation shows prediction accuracies of up to 98.2% and precisions up to 78.8% This indicates that speech analysis in combination with ML could be beneficial to identify suspected concussion cases.
Fedorchak et al. (2021)	Assess the ability of salivary non-coding ribonucleic acids (ncRNA) levels to predict post-concussion symptoms (PPCS).	*N* = 112 subjects with mTBI. Mixed sample (49F) and 82 (i.e., 73%) of mTBI are sports related. Age: 8–24 (M.age = 16). Sport: N.R. Region: North America	Cohort study and experimental design: the sample divided into two groups based on self-reported symptom scores (PPCS = 32, and non-PPCS = 80). Measures: medical/demographic characteristics via survey at enrolment, ncRNA collected ≤ 14 days post-injury, and follow-up ≥ 21 days post-injury. Balance and cognitive test performance were assessed at both these time-points. ML-method: a radial support vector machine (rSVM) algorithm.	The performance of model predicting PPCS status when measuring ncRNA within 14 days of concussion achieved an AUC of 0.83 and was superior to the modified clinical risk score (AUC= 0.73). Saliva ncRNAs biomarkers measured within 14 days of mTBI provide prognostic information about risk for PPCS, tracing recovery and predicting who will have prolonged symptoms.
Ferris et al. (2021)	Examine the validity of Vestibular/Ocular-Motor Screening (VOMS) assessment tool and utilize machine learning to deduce the additive power of the VOMS in relation to components of the Sport Concussion Assessment Tool 3 (SCAT3) for SRC detection.	*N* = 388 collegiate athletes (143 F and 245 M). Age: N.R. (mean = 19.1). Sports: N.R. Region: North America	Cohort-study; Data from National Collegiate Athletic Association–Department of Defense Concussion Assessment, Research and Education (CARE) Consortium. Measures: preseason and acute postinjury assessments including modified SCAT3 and VOMS. ML-method: an Ada Boosted Tree machine learning model.	Incorporation of VOMS into the full SCAT3 significantly boosted overall diagnostic ability by 4.4% (AUC = 0.848) and produced a 9% improvement in test sensitivity and 3% specificity over the existing SCAT3 battery. The results from this study highlight the utility of the VOMS tool in acute concussion assessments.
Gabler et al. (2020)	Develop and evaluate a broad range of ML model algorithms and predictive features for their ability to discriminate between head impacts and spurious events.	*N* = 21 collegiate athletes. Age: N.R. Sex: Male. Sport: football Region: North America	Cohort study over the course of 11 games during the fall 2018 and 2019 seasons. Measures; an instrumented mouthguard for measuring linear and angular head kinematics during impacts. Included video to verify and assessments of head impacts. Five different ML model algorithm classes were considered; Classification and Regression Tree (CART), Adaboost, XGBoost, The Random Forrest and Support Vector Machine (SVM).	All the five models revealed good performance filtering head impacts from spurious events on training dataset from 2018 season (precision = 91.7–95.9%). The results highlight the potential efficacy of the mouthguard sensor for detecting 81.6% of the head impacts confirmed on video, while the ML classifier achieved 100% recall at 98.3% precision. This indicates using sensor and ML model is promising for classifying head impacts in football.
Goswami et al. (2016)	Examine the uncinate fasciculus (UF) and connected gray matter in relation to behavioral changes in retried professional athletes with multiple concussions. Furthermore, to use ML to test the predictive power of diffusion imaging metrics within the UF to discriminate concussed athletes from controls.	*N* = 36. Two groups; (1) Retired male athletes with multiple concussion, *N* = 19. (2) Healthy males closely matched for age and education level with no history of or suspected concussions, *N* = 17. Age: 30–74 Sport: football Region: North America	Explorative design. Measures; neuropsychological assessments, neuroimaging (MRI) and cortical thickness analysis (CTA) to assess gray matter. ML-method: SVM classifiers	UF diffusion imaging differentiates athletes from healthy controls. These implicate the UF system in the pathological outcomes of repeated concussion as they relate to impulsive behavior. Furthermore, a SVM has potential utility in the general assessment and diagnosis of brain abnormalities following concussion.
Helfer et al. (2014)	Develop an easily obtainable biomarker for detecting cognitive change by using formant track dynamics and coordination.	*N* = 32 high school athletes; 25 male football players and 7 female soccer players. Age: 15–18. Sports: football, soccer. Region: North America	Cohort study; pre-, in- and postseason data. Measures; Multimodal Early Detection Interactive Classifier (MEDIC) system. Includes scores from a series of cognitive tests (ImPACT), along with speech features extracted from audio recordings from a standardized read passage. ML-method: A SVM-based classifier.	Findings demonstrate the use of vocal features during read speech to detect changes in cognitive ability. Detecting changes in cognitive status has potential benefit, in that cognitive changes have been shown to arise prior to clinically diagnosed concussions. Furthermore, the high detection rate of the classifier suggests it could be used as a screening tool to determine readiness to RTP thereby lowering the subject's risk of subsequent injury.
McNerny et al. (2019)	Evaluate the accuracy and potential benefit of including EEG measurements in a system that could provide an immediate objective mTBI assessment.	*N* = 85 (27 females, and 58 males). Two groups; (1) Subjects with a recent mTBI, N= 38 (2) Healthy control matched for age and sex with no history of mTBI within the last 5 years, *N* = 47. Age: 18–32. Sport: N.R. Region: North America	Questionnaire, behavioral tests and resting-state EEG using three frontopolar electrodes. ML-method: TotalBoost algorithm (analysis: leave-one-out-cross-validation).	The addition of EEG measurements boosted the accuracy to approximately 91 ± 2% compared to 82 ± 4% from the symptom questionnaire alone. This demonstrates the potential benefit of including EEG measurements to diagnose suspected mTBI. A step toward accurate and objective classification measurements that can be implemented on the field as a future injury assessment tool.
Raji et al. (2020)	Determine whether edge density imaging from MR can separate pediatric mTBI from typically developing controls.	*N* = 24. Two groups; (1) Subjects with a mTBI at least 1 month prior to enrolment, *N* = 14 (2) Typically developing controls matched in age, handedness and education, *N* = 10. Age: 10–16. Sex: N.R. Sports: 10 of 14 had sports-related causes (soccer, skiing, basketball, water polo and football). Region: North America	Experimental design. Measurements; neurocognitive assessments including the pediatric version of the California Verbal Learning Test (CVLT) and the Attention Network Task (ANT), and magnetic resonance imaging (MRI) scan/assessments. ML-method: Support vector machine (SVM) using linear kernels (analysis; ROC and leave-one-out cross-validation).	SVM-principal component analysis of edge density imaging maps identified three white matter regions distinguishing pediatric mild TBI from controls. This show that edge density imaging is a new form of connectome mapping that provides better diagnostic delineation between pediatric mild TBI and healthy controls than neurocognitive assessments of memory or attention.
Reynolds et al. (2018)	Use rs-fMRI data to assess effects of subconcussion on metrics thought to represent functional brain connectivity.	*N* = 72 males. Three groups; (1) High subconcussive exposure (CF), *N* = 15 football players. (2) Medium subconcussive exposure (OS), *N* = 28 soccer and lacrosse players (3) Low subconcussive exposure (MC), *N* = 29 controls. Age: N.R. (mean = 20.3) Sports: football, soccer, lacrosse. Region: North America	Cohort study; pre- and postseason testing. Measurements; resting-state functional magnetic resonance imaging (rs-fMRI) to assess changes in the brain. ML-method: A linear support vector machine (SVM) classifier.	The paired SVM only found significantly high-class accuracy for preseason-to-postseason ReHo changes in the college football players (87%, *p* = 0.009). This indicates that CF players' local functional connectivity changed over the course of a single season.
Seeger et al. (2020)	Examine the utility of salivary inflammatory markers following SRC to predict symptom burden and length of return to sport (RTS).	*N* = 36 athletes (29 males, and 7 females. Age: 12–17. Sports: ice hockey. Region: North America	Prospective exploratory cohort study. Measurements; saliva samples collected within 72 h of injury and analyzed for cytokines. In addition, participants' characteristics, length of RTP and symptom burden using SCAT3. ML-method: The RReliefF feature ranking algorithm.	The ML models used provided a specific cytokine profile in conjunction with sex and a previous concussion history that significantly correlated actual to predicted scores for the number of symptoms and symptom severity but not RTS. From these data, saliva cytokines hold promise as a method to identify fluid biomarker profiles for predicting symptom burden following SRC.
Shim et al. (2020)	Propose a framework that combines fine element (FE) analysis with a machine learning approach to simulate mTBI as a result of a direct impact to the head for rapid prediction of brain damage pattern after mTBI.	*N* = 33 males. Age: N.R. (mean = 20,3). Sport: football. Region: North America	Experiment using data from cohort study (pre-, during- and postseason). Measurements; magnetic resonance imaging (MRI) scan to develop a fine element model of the brain. ML-method: A Partial Least Squares Regression (PLSR) model using data from different brain impact scenarios.	The PLSR trained model was able to predict the general principal strain distribution patterns as well as the location and magnitude of peak strains with an accuracy of 95% and computational time of <10 s. This may play an important role in developing an objective diagnostic tool for mTBI that can predict the severity of head impact.
Thanjavur et al. (2021)	Develop a deep learning long short-term memory (LSTM)-based recurrent neural network that is able to distinguish between non-concussed and post-concussed adolescent athletes using samples of resting-state electroencephalography (EEG).	*N* = 62 male adolescents. Two groups; (1) Athletes who had suffered a SRC, *N* = 27. (2) Non-concussed and age-matched athletes, *N* = 35. Mean age: 14.1 Sports: N.R. Region: North America	Explorative design. Measurements; resting-state electroencephalography (EEG) recordings as input. ML-method: A deep learning long short-term memory (LSTM)-based recurrent neural network classifier (ConcNet).	The ConcNet classifier consistently identified concussions with an accuracy of > 90%. It correctly identified the concussed participants and misclassified only a small number of controls. This represents a promising first step toward the development of an easy-to-use, objective, brain-based, automatic classification of concussion at an individual level.
Tremblay et al. (2017)	Characterize the *in vivo* signature of remote sports concussions to provide an objective diagnostic tool.	*N* = 30 retired male university-level athletes. Two groups; (1) Subjects who sustained SRC in early adulthood, *N* = 15, (2) Subjects with no prior history of concussions, *N* = 15. Age: 51–75. Sports: ice hockey and football. Region: North America	Experimental design. Measurements; neuropsychological testing, genotyping, and multimodal neuroimaging evaluation. ML-methods: four different classifiers. A linear Support Vector Machine (SVM), A Step-wise Penalized Logistic Regression, a Random Forest and a LogitBoost.	ML classifiers trained to detect remote concussions achieved detection accuracies up to 90%.
Visscher et al. (2019)	Explore the use of ML for novel insights into the differences in phenotypes between patients with concussions based on objective vestibular and balance performance.	N= 96 subjects suspected of suffering from a SRC or PCS (78 males and 18 females). Age: N.R. Sports: ice hockey, ski, snowboard, handball, soccer. Region: Europe, Switzerland	Exploratory study. Measurements; balance and vestibular diagnostic testing, as well as epidemiological and symptoms data used for cluster analysis (*N* = 53 variables). ML-methods: Kohenn's self-organizing map (SOM).	The SOM divided the data into one group with prominent vestibular disorders and another with no clear vestibular or balance problems, suggesting that artificial intelligence might help improve the diagnostic process. This study could be helpful in the future for improving assessment batteries and diagnostic criteria.
Wu et al. (2019)	Develop a deep learning neural network to estimate reginal brain strains instantly and accurately using data from head impact sensors.	Real-world datasets (measured/ reconstructed) collected from head impact sensors (*N* = 3,069). Age: N.R. (high school, college and senior). Sex: N.R. Sports: football, boxing, mixed martial arts. Region: North America	Exploratory design. Measurements; head impact sensors converted into three regional brain strains. ML-method: A convolutional neural network (CNN) to convert a head impact measured (10-fold cross-validation).	The CNN estimated regional brain stains with sufficient accuracy. Together with sensors that measure impact kinematics, the CNN may enable a sophisticated head injury model to produce region-specific brain responses, instantly. Therefore, this technique may offer clinical diagnostic values to facilitate head impact sensors in concussion detection *via* a mobile device. This is important to mitigate the millions of concussion incidents worldwide every year.
Zhuang et al. (2021)	Simulation of force impacts with bowling bowl on a single FBG-embedded smart helmet prototype (football).	Development of new sports equipment (protective head gear).	Exploratory. Development of a smart helmet with a single embedded fiber Bragg grating (FBG) sensor for real-time sensing of blunt-force impact events to helmets. ML-method: 7 machine learning models were considered [Support Vector Machine (SVM), Gaussian Process Regression (GPR), Random Forest (RF), K-Nearest Neighbor Instance-Based Learner (IBK), Elastic Net Regression (ENR), Voting, and Additive Regression-Random Forest (AR-RF)].	The FBG-embedded smart helmet prototype successfully achieved real-time sensing of concussive events. The use of ML-FBG smart helmet systems can serve as an early-stage intervention strategy during and immediately following a concussive event.

### Machine Learning for the Prediction and Prevention of SRC

Of the 24 studies included in the review, 10 focus on the use of machine learning in predicting and preventing SRC (see Appendix 1). The studies in this management phase of SRC are diverse in terms of methodological approach, sample and aim. Three of the 10 studies focus on the role of biomarkers in the prediction of SRC (Helfer et al., 2014; Seeger et al., 2020; Fedorchak et al., 2021). For instance, Seeger et al. (2020) studied youth ice hockey participants (aged 12–17 years) to explore cytokine alterations following pediatric SRC. Their aim was to determine whether a specific cytokine profile could predict symptom burden and time to return to sports (RTS). Using saliva cytokine samples, they determined that there is evidence of alterations in saliva cytokine profiles that are associated with increased symptom burden following SRC. Seeger et al. (2020) therefore provide support for the feasibility of exploring alterations in salivary cytokines in adolescent athletes' post-SRC and the potential to associate increased symptom burden and recovery.

Other studies attempt to use machine learning to develop new prediction models for SRC (Bergeron et al., 2019; Gabler et al., 2020; Castellanos et al., 2021). For example, in their study, Castellanos et al. (2021) carried out baseline assessments of 15,682 participants and monitored them for SRC during the subsequent season. This data was then used to develop a risk-stratification tool capable of identifying athletes with an elevated risk of SRC. The tool is based on a predictive algorithm for SRC. Castellanos et al. (2021) contribution is clinically important because it represents the first successful attempt to predict SRC using baseline data from athletes.

Another notable exploratory contribution in this category of the reviewed literature is that of Zhuang et al. (2021), who use machine learning and simulation of force impacts to develop a smart helmet prototype with real-time sensors that record blunt-force impacts to the head. In their study, Zhuang et al. (2021) found that the smart helmet prototype successfully achieved a real-time sensing of concussive events and concluded that the novel smart helmet system could serve as an early-stage intervention strategy during a concussive event.

Overall, only one of the 10 studies categorized as prediction and prevention apply a deep learning approach (Domel et al., 2021), while the remaining 9 are conducted using machine learning algorithms, such as RReliefF (Seeger et al., 2020), XGBoost (DiCesare et al., 2020), Support Vector Machine (SVM) (Helfer et al., 2014; Reynolds et al., 2018) and Adaboost (Gabler et al., 2020).

### Machine Learning and the Diagnosis and Classification of SRC

Eleven of the 24 studies included in the review examine how machine learning can be used in the diagnosis and classification of SRC (see Appendix 1). Several studies are devoted to using machine learning to create tools that classify SRC more accurately than the current approaches (Cai et al., 2018; Visscher et al., 2019; Bazarian et al., 2021; Ferris et al., 2021). Some studies also attempt to develop novel diagnostic tools to rapidly and objectively detect SRC (Tremblay et al., 2017; McNerny et al., 2019; Shim et al., 2020). One example is the study conducted by McNerny et al. (2019), which uses a unique combination of simple EEG measurements paired with advanced quantitative analysis in order to develop a highly accurate injury assessment tool. Studies such as those by Tremblay et al. (2017); McNerny et al. (2019) and Shim et al. (2020) represent valuable contributions to the field, in that developing tools that facilitate an accurate diagnosis of SRC is of the utmost importance, especially as many athletes and coaches under-report symptoms and “believe it is fine to play through a concussion even with background knowledge of the potential dangers” (McNerny et al., 2019, p. 6). The metabolic crisis that occurs in the brain following a concussion makes the brain vulnerable to subsequent injuries that could result in chronic brain disease. As McNerny et al. (2019, p. 6) highlight: “the period of vulnerability is unknown as it depends on many factors, such as severity, but it is clear that a subsequent injury occurring prior to full physiological recovery can be detrimental to the brain.” Therefore, it is important to create a way of measuring the initial injury and the recovery of that injury.

Two of the studies on the diagnosis and classification of SRC engage in deep learning approaches (Wu et al., 2019; Thanjavur et al., 2021). In their study, Wu et al. (2019) developed a trained neural network that is capable of assimilating impact-response samples to iteratively improve accuracy. Together with sensors that measure the impact of kinematics on head collisions, Wu et al. (2019) technique may enable a sophisticated head injury model to produce region-specific brain responses. Wu et al. (2019) and Thanjavur et al. (2021) studies showcase novel techniques that may offer clinical diagnostic values to SRC, and in Wu et al. (2019) case, even facilitate head impact sensors in concussion detection via a mobile device. This could be a valuable way of mitigating the millions of SRC incidents worldwide every year. The remaining 9 studies [excluding those of Wu et al. (2019) and Thanjavur et al. (2021)] are based on machine learning algorithms, such as TotalBoost (McNerny et al., 2019), Support Vector Machine (SVM) (Falcone et al., 2013; Tremblay et al., 2017; Raji et al., 2020), or Kohenn's Self-Organizing Map (SOM) (Visscher et al., 2019).

### Machine Learning in the Treatment and Rehabilitation of SRC

Only 3 of the 24 analyzed studies focus on how machine learning can contribute to the treatment and rehabilitation of SRC (Cao et al., 2008; Goswami et al., 2016; Bohsra et al., 2019). Both Cao et al. (2008) and Goswami et al. (2016) investigated brain abnormalities following concussive episodes and applied a SVM classifier to train and test multichannel EEG (Cao et al., 2008) or rs-fMRI (Goswami et al., 2016) data. Their findings, which are promising, could help clinicians with more accurate return-to-sport participation criteria (Cao et al., 2008) or to predict recovery following a SRC event (Goswami et al., 2016).

Bohsra et al. (2019) demonstrate the use of machine learning as a methodological approach to utilizing the neurophysiological markers found in group-level studies relating to the detection of SRC in individual athletes. They identify a set of features that can help to accurately detect past concussion in up to 81% of retired athletes. Through this machine learning approach, Bohsra et al., 2019 were able to correctly and accurately classify past concussion even though the concussed athlete received their last injury up to “45 years ago, which is far beyond the suggested time frame of a few months for symptom resolution” (Bohsra et al., 2019 p. 1498). Bohsra et al. (2019) represents a first report of machine learning based analysis for the assessment of SRC history decades after injury, which is a significant step in the identification of chronic effects of concussion in athletes and an important factor in the rehabilitation and treatment of long-term health issues following SRC.

Both Bohsra et al. (2019) and Goswami et al. (2016) findings have potential applications for treatment and rehabilitation, as well as for the diagnosis and classification of SRC. As Bohsra et al., 2019 ( p. 1498) state, their results: “can be used by health professionals if machine learning tools like the one described here are adopted as diagnostic aids in the future.” Goswami et al. (2016, p. 10) also highlight that their work suggests “the potential utility of machine learning for the diagnosis of brain injury following concussions.”

## Discussion

The results of this scoping review suggest that although, the use of machine learning for athlete injury prevention includes a plethora of studies (Juhiainen et al., 2021; Van Eetvelde et al., 2021), machine learning and the management of SRC is a novel and somewhat underdeveloped research field. Most of the studies in the review focus on predicting and preventing SRC (*n* = 10) or diagnosing and classifying SRC (*n* = 11), while only 3 studies explore the treatment and rehabilitation of SRC (see Table 1 and Appendix 1). From an athlete welfare perspective this makes sense, as one would prefer to prevent SRC from ever happening, especially in youth sports. The focus on developing accurate tools that coaches and athletes can use to diagnose SRC appears to be especially important, as several studies highlight the lack of objective tools to measure and classify SRC symptoms rapidly (Shim et al., 2020; Thanjavur et al., 2021).

The discussion is guided by the second purpose presented for this scoping review in the introduction, which is to ascertain where there are gaps in the existing research and identify recommendations for future research. Therefore, we first present an overview of the empirical study characteristics of the reviewed literature (who is the research on machine learning for the management of SRC based on?), and secondly identify knowledge gaps and the need for future research.

### Study Context and Sample: Who Is the Research on Machine Learning in the Management of SRC Based on?

Current research on machine learning and the management of SRC is mainly conduced on male, adolescent or adult athletes from team sports in North America. As Table 2 demonstrates, only one article has a female only sample. This is DiCesare et al. (2020) study of female soccer players in North America. While some studies have gender mixed samples, many of these report a predominately male sample. For instance, Seeger et al. (2020) report a sample of 29 male athletes and 7 female athletes. The absence of studies on female athletes in research on machine learning in the management of SRC reflects the sex data gap in sport and exercise science research field. In a recent meta-study of medical and physiological sport and exercise research, Cowley et al. (2021) found that out of 5,261 publications and 12,511,386 participants, 63% of publications included both male and female participants, 31% included males only, and 6% included females only. In these studies there was a total of 8,253,236 (66%) male participants and 4,254,445 (34%) female participants, across all journals (Cowley et al., 2021). In other words, while women are included as participants in sport science research generally, and SRC injury research specifically, the numbers of female participants are not equal to the quantity of male participants. The sex data gap does, however, appear to be more extensive in studies of machine learning for the management of SRC than in the sport and exercise field generally (Cowley et al., 2021). This may be explained by the relatively small number of machine learning studies in the SRC research field (*n* = 24).

**Table 2:** Study characteristics of the reviewed literature.

		***N***
Athlete gender	Female	1
	Male	8
	Mixed	10
	Not reported	5
Athlete age	Child (3–5)	-
	Youth (6–11)	-
	Adolescent (12–18)	5
	Adult (19+)	4
	Mixed youth and adolescent	-
	Mixed adolescent and adult	3
	Mixed youth, adolescent and adult	2
	Not reported	10
Sports	Boxing	1
	Football	7
	Ice hockey	1
	Soccer	1
	Mixed	11
	Not reported	3
Region sample is recruited from	North America	22
	Europe	1
	Not reported	1
Sample size	<20	-
	20–50	10
	51–100	5
	101–200	2
	201–500	1
	>501	2
	Not reported	4

In terms of age, none of the studies in this scoping review report a sample of children (age 3–5) or youth (6–11) athletes. Five studies use data from adolescent athletes (age 12–18) and four studies report on adult athlete samples (see Tables 1, 2). However, 10 studies do not report the age of the athletes in the sample at all (e.g., Falcone et al., 2013) and/or provide insufficient information about age. For instance, Visscher et al. (2019) report the median age, while Ferris et al. (2021) present the mean age in their samples. This variability in the quality of reporting an important variable such as age can create problems for the generalisability of the findings.

Team sports dominate the data of the analyzed studies. These sports include American football, ice hockey, soccer, basketball, lacrosse, rugby, volleyball, water polo and handball. The most prominent team sport is American football, with seven studies dedicated to this sport (see Table 2), as well as being included in 9 of 11 studies with mixed sports (Cao et al., 2008; Helfer et al., 2014; Tremblay et al., 2017; Reynolds et al., 2018; Bergeron et al., 2019; Wu et al., 2019; Raji et al., 2020; Bazarian et al., 2021; Castellanos et al., 2021). While many individual sports are included in the mixed samples, such as track and field (Helfer et al., 2014; Castellanos et al., 2021), mixed martial arts (Wu et al., 2019), snowboard (Visscher et al., 2019), skiing (Raji et al., 2020), tennis (Castellanos et al., 2021) and wrestling (Bergeron et al., 2019), there is only one individual sport with its own study: boxing (Falcone et al., 2013). In the studies with data from a mix of sports, it is often uncertain how many athletes from each sport are included.

Twenty-two of 24 studies included in the review have been conducted in North America (see Table 2). It is not uncommon for research fields to be predominately based in the Global North. Hence, we were not surprised to find that this is also the case for the machine learning and SRC field. Still, we were perhaps expecting more contributions from Europe. Only one study in our review is conducted in a European sports context (Visscher et al., 2019). Lastly, most of the studies in the review report relatively small samples of athletes. Two of the 24 studies report a sample of more than 501 athletes (see Table 2), and although no studies report <20 athletes, 10 studies have samples of between 20 and 50 athletes. This seems somewhat paradoxical, as the advantage of machine learning is that it can analyse large, complex and unstructured data and reveal patterns, trends and associations that traditional methodological approaches cannot (Agoston, 2019).

### Publication Frequency and Scholarly Discipline

There has been a dramatic increase in articles published on the potential of machine learning for the management of SRC. As seen in Table 3, there is a large increase in the number of articles published from 2016 and onwards. Only one article was published before 2011 (Cao et al., 2008) and only two were published between 2012 and 2015 (Falcone et al., 2013; Helfer et al., 2014). Looking at the scholarly field represented in these studies, Table 3 shows that research on machine learning and SRC is mainly published in engineering and medical journals. Overall, six articles are published in engineering journals, seven articles in general academic journals, and the remaining 11 articles in medical journals.

**Table 3:** Reviewed literature by journal and year of publication.

**Journal**	**2008–2011**	**2012–2015**	**2016–2019**	**2020–2021**	***N***
Annals of Biomedical Engineering				2	2
Brain Imaging and Behavior			1		1
Brain Structure and Function			1		1
European Journal of Neuroscience			1		1
IEEE Access				1	1
IEEE International Conference on Acoustics, Speech, and Signal Processing		1			1
IEEE Transactions on Neural Systems and Rehabilitation Engineering	1		1		2
ISCA Proceedings of Interspeech		1			1
JAMA Network Open				1	1
Journal of Neurology				1	1
Journal of Neuroscience Methods				1	1
Medicine & Science in Sports & Exercise			1		1
Pediatric Radiology				1	1
PLoS ONE			2		2
Scientific Reports			1	2	3
Sports Medicine				1	1
Sports Medicine - Open			1		1
The American Journal of Sports Medicine				1	1
The Journal of Head Trauma Rehabilitation				1	1

### Research Agenda and Current Knowledge Gaps

The use of machine learning for the management of SRC is a relatively new area of research. The surge in publication frequency on this topic since 2016 (see Table 3) indicates that this is a growing yet still under-explored research field. The potential of machine learning for the prevention, classification and treatment of SRC is still unclear. A main finding from our scoping review is that new methods of machine learning analysis for the management of SRC are now being actively explored in engineering and medical research. The small number of published papers on the use of machine learning in the management of SRC (*n* = 24) could also suggest that its value is not yet apparent to the research community on safeguarding in sports. From our review, it would seem that the potential value of employing machine learning in the management of SRC is still inconclusive.

As the field is novel and there is limited research on the topic, pointing to clear directions for future research is challenging. Nevertheless, we have three recommendations to make based on our analysis of the 24 articles included in this review. These are: (1) more investigations into the potential of deep learning, (2) more diverse data sets to be analyzed by machine learning algorithms and (3) a need for social scientific perspectives on the implementation of novel technologies and tools created through machine learning approaches.

The review demonstrates that there are almost no studies on the treatment and rehabilitation of SRC and that there are only a few select studies on the deep learning and management of SRC. As Cai et al. (2018, p. 16) note: “the superior performances of deep learning in concussion prediction and suggest its promise for future applications in biomechanical investigations of traumatic brain injury.” Still, it is worth noting that our search string has been specifically focused on machine learning and that there may be other research contributions on deep learning that have not been identified in this scoping review. Furthermore, the number of studies on evidence-based treatment and rehabilitation for SRC remains sparse, which might explain why there are so few studies on machine learning in the management of SRC that addressing this topic.

Secondly, there is a lack of diversity in the samples in the reviewed literature. A prominent limitation in current research is the lack of empirical samples from sporting contexts outside North America. As far as we can ascertain, no studies have been conducted with data from athletes in Asia, Africa, South America or Oceania, and there are almost no studies with data from Europe. This does not mean that there is no research on machine learning and the management of SRC from these continents, but could simply mean that it is not currently reported in English. Furthermore, there is a need for more investigations with data from female athletes. The lack of studies on female athletes is an issue that has been identified in other reviews of the SRC field (Tjønndal et al., 2022). Additionally, many of the studies focus on team sports, so there is less knowledge about machine learning and SRC in individual sports. In other words, there is a need for diversity in the athlete data analyzed by the machine learning algorithms. Otherwise, the findings from this research field will lose credibility among athletes and coaches over time.

Finally, the review has revealed that there are no social scientific investigations into machine learning and the management of SRC. As knowledge about the potential applications of machine learning and deep learning for the prevention, diagnosis and treatment of SRC progresses, there will be a salient need for social science perspectives in this research field. For instance, many of the studies included in the review focus on the development of practical tools for the different management phases of SRC. For instance, Wu et al. (2019) estimate that the practical implication of their machine learning technique may offer clinical diagnostic values that could facilitate head impact sensors in concussion detection via a mobile device. Zhuang et al. (2021) hope to develop a new type of sporting equipment - a smart helmet. Both these examples require athletes and coaches to embrace the use of novel technologies and to manage them effectively. However, current social scientific studies of athletes' and coaches' experiences of new sport technologies has demonstrated that athletes and coaches are often dissatisfied with the usability of new sports technologies and feel that such technologies do not meet their needs (Trabal, 2008; Luczak et al., 2020). Machine learning approaches to the management of SRC might also entail new ethical issues related to coaching athletes. While machine learning could bring new advances in terms of predicting the risk of athletes contracting SRC, when should coaches draw the line and take athletes off the field? Based on predictions from the machine learning algorithms of previous SRC events, a coach is likely to know when their athletes are at risk of serious injury. However, as coaches only keep their job if they can provide results and win competitions, the threshold for taking a star athlete out of an important game is likely to be high. With potential issues like these, social scientific investigations will be needed in the future, in order to understand what athletes and coaches need from novel procedures, equipment and tools based on machine learning approaches for the management of SRC.

## Conclusion

The purpose of this review has been 2-fold: (1) to summarize and map the existing research literature on the use of machine learning in the management of SRC and (2) to ascertain where there are knowledge gaps in the existing research. This review concludes that most studies on machine learning and the management of SRC have either focused on the diagnosis and classification of SRC, or on the prediction and prevention of SRC. Very few studies on the treatment and rehabilitation of SRC have been identified in this review. The limited number of studies on machine learning in the treatment and rehabilitation of SRC might be explained by the broader lack of evidence-based studies on treatment and rehabilitation for SRC.

In the prediction and prevention category, studies are divided in two overarching categories. Some studies explore the role of biomarkers in predicting and preventing SRC, while others work toward the development of new prediction models for SRC based on various machine learning approaches and datasets. Contributions to the diagnosis and classification of SRC are mainly devoted to developing novel diagnostic tools to detect and classify SRC rapidly and more accurately than the currently available diagnostic approaches.

Most of the reviewed studies employ machine learning algorithms to analyse athlete injury data derived from male athletes playing team sports in North America. As Zhang et al. (2020, p. 1956) argue: “There is urgency for sex and gender sensitive concussion research, as well as a need to develop better and more accurate care strategies for wider demographics.” From our review, it would appear that this also applies to future research on the potential of machine learning for the management of SRC. Research on machine learning in the management of SRC mirrors the current sex data gap in sports and exercise research (Cowley et al., 2021). However, such narrow empirical data, at least in some cases, makes us question the transferability and usability of these findings. There is also a need for social science investigations of the usability and implementation of new tools and sports equipment aimed at athletes and coaches, given that previous studies indicate that new sports technology is often met with resistance and skepticism by practitioners (Trabal, 2008; Luczak et al., 2020).

Further studies using more diverse athlete data for analysis through machine learning algorithms may have the potential to fill important knowledge gaps in safeguarding and preventing injury. Addressing these gaps may also improve athletes' welfare by reducing the risk of sustaining SRC, as well as provide better treatment and rehabilitation from SRC. This review indicates that there could be potential in exploring the use of machine learning in the management of SRC. However, if the datasets used in this research continue to be based on narrow samples of athletes, there are considerable risks associated with the development of any new diagnostic and predictive tools for SRC emerging from this research. Today these risks appear to mainly affect the health and safety of female athletes of all ages. More diverse athlete data is thus needed to ensure that the development of such tools does not have unintended negative consequences for the health and safety of female athletes around the world.

## Author Contributions

All authors listed have made a substantial, direct, and intellectual contribution to the work and approved it for publication.

## Funding

Nord University funded the open access publication of this article.

## Conflict of Interest

The authors declare that the research was conducted in the absence of any commercial or financial relationships that could be construed as a potential conflict of interest.

## Publisher's Note

All claims expressed in this article are solely those of the authors and do not necessarily represent those of their affiliated organizations, or those of the publisher, the editors and the reviewers. Any product that may be evaluated in this article, or claim that may be made by its manufacturer, is not guaranteed or endorsed by the publisher.
