# Supplementary material for: Safeguarding Athletes Against Head Injuries Through Advances in Technology: A Scoping Review of the Uses of Machine Learning in the Management of Sports-Related Concussion
Source: Front Sports Act Living. 2022 Apr 20;4:837643. doi: 10.3389/fspor.2022.837643 (PMC9067303; doi:10.3389/fspor.2022.837643)
Supplement: Supplementary file 1 [file Table_1.docx]

Appendix 1: Reviewed literature categorised by management phase

| **Management phase** | **Articles** |
| --- | --- |
| Diagnosis and classification | Bazarian et al. (2021)  Cai et al. (2018)  Falcone et al. (2013)  Ferris et al. (2021)  McNerney et al. (2019)  Raji et al. (2020)  Shim et al. (2020)  Thanjavur et al. (2021)  Tremblay et al. (2017)  Visscher et al. (2019)  Wu et al. (2019) |
| Prediction and prevention | Bergeron et al. (2019)  Castellanos et al. (2021)  DiCesare et al. (2020)  Domel et al. (2021)  Fedorchack et al. (2021)  Gabler et al. (2020)  Helfer et al. (2014)  Reynolds et al. (2017)  Seeger et al. (2020)  Zhuang et al. (2021) |
| Treatment and rehabilitation | Boshra et al. (2019).  Cao et al. (2008)  Goswami et al. (2016) |
